# Pro-Aging Effects of Xanthine Oxidoreductase Products

**DOI:** 10.3390/antiox9090839

**Published:** 2020-09-08

**Authors:** Maria Giulia Battelli, Massimo Bortolotti, Andrea Bolognesi, Letizia Polito

**Affiliations:** Department of Experimental, Diagnostic and Specialty Medicine-DIMES, Alma Mater Studiorum, University of Bologna, Via San Giacomo 14, 40126 Bologna, Italy; mariagiulia.battelli@unibo.it (M.G.B.); massimo.bortolotti2@unibo.it (M.B.)

**Keywords:** aging, antioxidants, oxi-inflamm-aging, oxidative stress, xanthine oxidoreductase

## Abstract

The senescence process is the result of a series of factors that start from the genetic constitution interacting with epigenetic modifications induced by endogenous and environmental causes and that lead to a progressive deterioration at the cellular and functional levels. One of the main causes of aging is oxidative stress deriving from the imbalance between the production of reactive oxygen (ROS) and nitrogen (RNS) species and their scavenging through antioxidants. Xanthine oxidoreductase (XOR) activities produce uric acid, as well as reactive oxygen and nitrogen species, which all may be relevant to such equilibrium. This review analyzes XOR activity through in vitro experiments, animal studies and clinical reports, which highlight the pro-aging effects of XOR products. However, XOR activity contributes to a regular level of ROS and RNS, which appears essential for the proper functioning of many physiological pathways. This discourages the use of therapies with XOR inhibitors, unless symptomatic hyperuricemia is present.

## 1. Introduction

Over the last 70 years, life expectancy has grown progressively, particularly in industrialized countries, due to improved living conditions and advances in medical science. As a consequence, the share of the elderly in the population is continuously increasing, and with it the diseases associated with aging [1]. Population aging and the related morbidity is becoming not only a public health concern but also a socio-economic problem at least in the Western world, thus stimulating ever more research in this field.

Life is characterized by a continuous modification of both molecules and cells that are the living material constituents. This biological “Panta rhei” inexorably leads to senescence through a multi-etiological process that progressively impairs the body functions. This article has the aim of analyzing the contribution of xanthine oxidoreductase (XOR) activities and products to the aging process.

To this purpose, we start by revising the consequences of oxidative stress resulting from the imbalance between oxidant factors and antioxidant physiological components in relationship to senescence. Then, we examine the structure and the activities of XOR taking in consideration the products of such activities and evaluating their pro- and antioxidant effects, especially those of nitric oxide (NO) and reactive oxygen (ROS) and nitrogen species (RNS). Finally, we analyze the relationship between XOR activity and aging by reviewing the pro-aging effects that are related to XOR products in in vitro experiments, animal studies and clinical reports, as detailed in Section 4. A series of in vitro experiments suggest a role for XOR-derived ROS in skin photo-aging, in the aging of osteoprogenitor cells and in advanced glycation end-products (AGE) formation in diabetic patients, as well as in degenerative cerebral pathologies, although with an ambivalent role. In aged rats, XOR-derived ROS influence the repair of injured nerves and generate oxidative stress in the heart, kidney, aorta and mesenteric arteries. In mice, XOR expression and activity increase with age in many organs with consequent oxidative stress, which in hematopoietic stem cells is associated with a higher level of DNA damage and apoptosis, a significantly shorter telomere length, a lower expression of plasticity markers and reduced activity of antioxidant enzymes. In addition, the increment of XOR-derived oxidative stress with age contributes to immunosenescence, as well as to higher oxi-inflamm-aging parameters. Clinical studies suggest that XOR activity, in old subjects, is implicated in the alteration of arteries dilation, in chronic cardiac failure, in kidney disease-related hypertension and in sarcopenia. Moreover, the increased XOR activity due to hyperglycemia could accelerate the onset of cataracts in senescence. However, the role as second messengers and the consequent ambivalent behavior of some XOR-derived ROS and RNS, as well as of uric acid, deter the an anti-age use of XOR inhibitors.

## 2. Oxidative Stress and Aging

**Figure 1:**
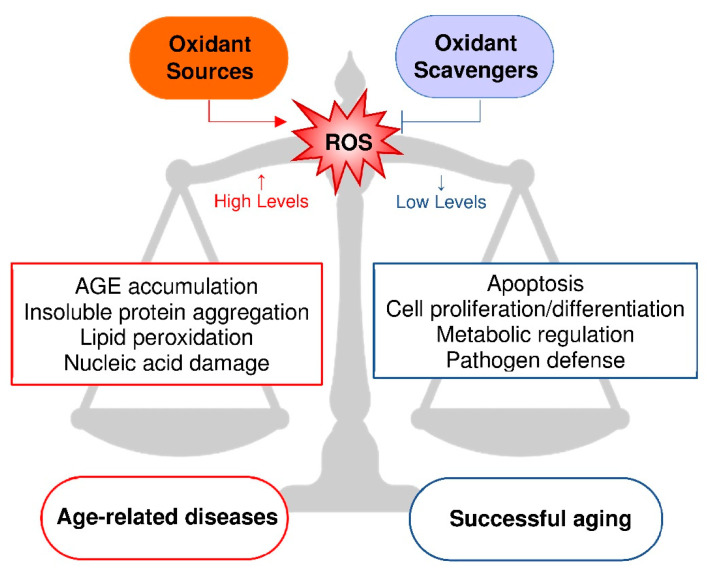
Relationship between reactive oxygen species (ROS) production and aging. The balance between oxidant sources and oxidant scavengers gives rise to pathologically high or physiologically low levels of ROS. High levels of ROS induce oxidative stress, leading to advanced glycation end-products (AGE) accumulation [20], insoluble protein aggregation [21], lipid peroxidation [22] and nucleic acid damage [23]. Low levels of ROS activate cellular signaling, such as apoptosis [24], cell proliferation and differentiation [25], metabolic regulation [26] and pathogen defense [27]. High or low levels of ROS cause age-related diseases or successful aging, respectively.

Oxidative stress occurs when the physiological redox systems fail to neutralize the reactive oxidant species produced by metabolism or deriving from exogenous sources, which therefore alter the biological molecules with consequent advanced glycation end-products (AGE) accumulation, oxidation of protein thiol groups, peroxidation of lipids and damage to DNA (Figure 1). The accumulation of molecular modifications induces a progressive cellular change which is, in turn, responsible for the decreased physiological capacities and increased morbidity of aging. Human aging is associated with a pro-oxidant state at least in part due to a reduction in non-enzymatic antioxidant components that determines a decrease in antioxidant capacities. Centenarians represent an exception, since this group of successfully aged people is characterized by an antioxidant profile that is associated with a conserved correct immunological response and with the absence of age-related diseases [2].

The fact that senescence is characterized by an imbalance between free radical production and antioxidant capacity supports the free radical theory of aging [3]. Accordingly, an animal model of dietary restriction was proposed as an anti-aging intervention, because it is able to extend the life span by limiting the metabolic rate with mitochondrial ROS production and consequent oxidative stress [4]. On the other hand, caloric restriction, as well as physical exercise, induces mitochondrial activation, thus augmenting the metabolic rate, oxygen consumption and heat production. The resulting increased formation of ROS could act as a signal by activating genes that cause an adaptive response (mitohormesis), which increases the antioxidant capacity and promotes longevity. Thus, ROS can have an opposite outcome depending on the amount produced, showing anti- or pro-aging effects at low or high levels, respectively [5,6].

Correct functioning of the immune system is a longevity predictor, and this is at the base of the immune theory of aging. The age-related dysregulation of the immune response is associated with a pro-phlogistic state, known as inflamm-aging [7], which can be aggravated by an inflammatory response related to metabolic stress, called metaflammation [8]. Both processes are mediated by the activation of the NLRP3 inflammasome [9]. In addition, the oxidative stress and pro-phlogistic state reported in senescence have been named oxi-inflamm-aging and contribute to the increased susceptibility of aged subjects to infectious diseases [10,11].

Beside mitochondria, endogenous sources of ROS are various oxidases [12], including XOR, whose activity produces ROS, NO and RNS both in physiological and pathological conditions [13]. Although alteration in redox signaling is associated with aging and disease, these reactive species, as shown in Figure 1, are involved in the modulation of transcription, DNA synthesis and repair, cell adhesion, apoptosis and autophagy by providing physiologically relevant signaling [14]. The oxidative stress-induced alteration of lipids, proteins and DNA somewhat unifies the aging theories based on free radicals with those related to mitochondrial changes, somatic mutations, accumulation of aberrant proteins, alteration of immunological responses and chronic inflammation [15,16].

The lipid peroxidation process originates toxic and mutagenic aldehydes, which can form stable adducts with cellular macromolecules and are considered markers of oxidative stress [1]. Aldehydes and their adducts augment with aging and are implicated in cancerogenesis and age-related neurodegenerative disorders, specifically Alzheimer’s and Parkinson’s diseases. In the latter, the oxidative damage to proteins can contribute to nigral cell death. The peroxidative damage to membranes can promote mitochondrial dysfunction and induce apoptosis. In addition, the oxidative DNA damage, including that to mitochondrial DNA, caused by an increased metabolic rate, has been associated with the shorter life span observed in smaller mammals [12].

A reduction in nitric oxide bioavailability and the consequent vascular endothelial dysfunction develop in aging even in healthy subjects, because of an increased activity of oxidative enzymes without an appropriate increase in antioxidant defenses. In addition, senescence induces genomic instability and contributes to endothelial oxidative stress and inflammation by activating transcription factors such as NF-κB that cause the transcription of pro-inflammatory cytokines [17].

Oxidative stress has a negative effect on multipotent bone marrow stromal cells by inhibiting their Hedgehog signaling-mediated osteogenic differentiation [18]. Oxidative stress-related senescence also affects stem cells by impairing their ability to repair injured tissues. Excessive ROS production or an insufficient antioxidant shield decrease stem cell efficiency, ending by reducing their number. In turn, stem cell depletion accelerates the aging process of the organism. In addition, the inflammatory cascade is elicited by oxidative stress through the activation of NF-κB, and the inhibition of NF-κB alleviates the consequence to the cell of oxidative stress by protecting staminal cells and reducing cell death in aged tissues [19].

Furthermore, aging is characterized by sarcopenia consisting of the loss of motor units, individual muscle fibers and muscle bulk, leading to a deficit in force generation at least in part due to the accumulation of products of oxidative damage [28]. Although the oxidative stress induced by an increase in ROS and RNS levels and a decrease in antioxidant defenses is recognized as responsible for the sarcopenic phenotype in the elderly, the attempt to combat sarcopenia and improve muscle strength through dietary supplementation with antioxidants gives controversial results [29].

## 3. Reactive Species Produced by XOR Activity

**Figure 2:**
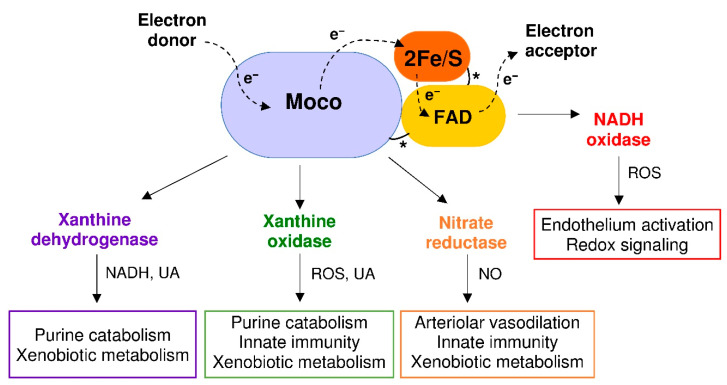
Xanthine oxidoreductase (XOR) structure and activities. Each XOR subunit is composed of three domains connected by unstructured regions (*) and characterized by: (i) two iron-sulfur redox centers (2Fe/S), (ii) a flavin adenine dinucleotide (FAD) cofactor and (iii) a molybdenum containing molybdopterin cofactor (Moco). The dotted arrows show the electron (e^−^) flux direction. The products and functions of each XOR activity are indicated: reduced nicotinamide adenine dinucleotide (NADH); nitric oxide (NO); reactive oxygen species (ROS); and uric acid (UA).

XOR belongs to a family of molybdenum-flavin iron-sulfur hydroxylases, which are widely distributed in living organisms from prokaryotic to eukaryotic species. In vertebrates, XOR has a homodimeric structure of about 290 kDa and each subunit is composed of three domains: a 20-kDa N-terminal domain with two non-identical iron-sulfur redox centers, a 40-kDa intermediate domain with a flavin adenine dinucleotide (FAD) cofactor and an 85-kDa C-terminal domain with a cofactor consisting of a molybdenum atom bound to a molybdopterin molecule (Moco). The domains are connected by means of unstructured hinge regions. The Moco-containing domain holds the substrate pocket, where oxidation occurs and from which the electron flux moves through the two iron-sulfur clusters towards the FAD-containing domain, where the electrons acceptor is reduced [30] (Figure 2).

In lower organisms, XOR has only a dehydrogenase activity, while in mammals the constitutive NAD^+^-dependent xanthine dehydrogenase (XDH, EC 1.17.1.4) can be converted to xanthine oxidase (XO, EC 1.17.3.2) by limited proteolysis or oxidation of sulfhydryl groups [31]. The transition of XDH to XO includes an intermediate XOR form that has both dehydrogenase and oxidase activity [31]. In the highest primates, XOR catalyzes the last two steps of purine catabolism, i.e., the oxidation of hypoxanthine and xanthine to uric acid, because mutations during evolution caused the loss of uricase activity. As a consequence, the uricemia is higher in uricotelic than in ureotelic animals. Many advantages have been suggested for this evolution, such as increased life expectancy due to the antioxidant action of circulating uric acid and its anti-cancer effect, at least when uricemia is in the normal range. When the mutations occurred, survival advantages derived also from the support given by uricemia to the blood pressure and to the accumulation of lipids, in a time in which dietary salt and meat were not always available [32]. Since uric acid is an irreversible product, XOR activity precludes the salvage pathway of purine nucleotides. In addition to catabolizing the purines, XOR metabolizes various endogenous and exogenous substrates, including some drugs. In human tissues, XOR is expressed at a high level only by epithelial cells of the lactating mammary gland, liver, intestine and kidney, and the dehydrogenase activity is prevalent within these cells. Instead, the oxidase form is associated with endothelial cells and XO activity is mainly present in biological fluids such as plasma and milk [33].

Uric acid produced by XOR activity, at the physiological level, helps to support a normal blood pressure and has an extracellular antioxidant action, which is protective against neoplastic transformation. On the other hand, uric acid and the free radicals derived from its reaction with ROS, NO and RNS may exert a pro-oxidant action, mostly inside the cell. When released from dead cells, uric acid behaves as a danger-associated molecular pattern and has a proinflammatory activity. Hyperuricemia contributes to endothelial dysfunction, hypertension, renal and cardiovascular diseases, as well as fatty liver, obesity, insulin resistance, diabetes and metabolic syndrome [32,34].

By delivering electrons directly to the molecular oxygen, XO generates a superoxide anion (O_2_**^•^**^−^) via a one-electron reduction and hydrogen peroxide (H_2_O_2_) via a two-electron reduction. These ROS, in turn, can produce hydroxyl radicals in the presence of iron or other transition metals through Haber–Weiss and Fenton reactions. XDH may produce O_2_^•−^and H_2_O_2_ at the FAD site by oxidizing NADH, especially under hypoxic conditions. In addition, XOR can reduce nitrates to nitrites and to NO, which generates peroxynitrite (ONOO^−^) and other RNS by reacting with ROS, thereby concurring with oxidative stress. On these bases, the cytotoxic action of XOR contributes to the microbicidal activity of the immune system as well as to the cell and tissue damage during ischemia/reperfusion injury. Furthermore, the killing ability of XOR products has been exploited for the production of immunoconjugates with the purpose of eliminating undesired cells [35]. The nitrate reductase activity of XOR occurs at the Moco site, as purine oxidative hydroxylation. Allopurinol and febuxostat inhibit both activities in a competitive or non-competitive way, respectively, but are ineffective toward the NADH-oxidizing activity of XOR [13].

In pathological conditions, XOR products are responsible for cytotoxicity and tissue damage or may promote mutagenesis, cell proliferation and tumor progression as well as endothelial dysfunction, in turn leading to atherosclerosis and cardiovascular diseases. In addition, XOR-derived oxidative stress and hyperuricemia promote metabolic alterations, thus contributing to various age-related diseases [34,36]. On the other hand, XOR products may activate apoptosis or cell differentiation pathways [37]. Moreover, XO-generated ROS contribute to innate immunity and are upstream to the signaling pathway leading to mitochondrial ROS production that is indispensable for the activation of the macrophagic NLRP3 inflammasome by pathogen-associated molecular patterns (PAMPs), damage-associated molecular patterns (DAMPs) or environmental irritants and consequent IL-1β secretion [38]. In addition, the reactive species produced by XOR exert a physiological role by activating the endothelial response in inflammation and they also have a redox signaling function resulting as essential in the regulation of arteriolar tone [36]. All these findings are in agreement with the reported role of ROS on several signaling pathways showing how XOR-generated ROS mediate cellular signaling [39].

## 4. XOR-Deriving Reactive Species and Aging

### 4.1. Molecular Pathways and Pharmacological Agents

Several pathways have been described to regulate aging. Notably, an extension in lifespan can be reached through both upregulation of sirtuin-1 (SIRT1), and downregulation of PI3K–AKT–mTOR and of the Insulin/Insulin-like Growth Factor (IGF) signaling pathways [40]. The main interconnection node between these pathways is represented by AMP-activated protein kinase (AMPK). For instance, under starved conditions, the activation of AMPK alters intracellular metabolism, culminating in an increase in NAD^+^ levels, with a concomitant increase in SIRT1 activity and downregulation of the mTOR and IF/IGF pathways [40]. These pathways can also regulate the aging process at different levels, for example, in mammals SIRT1 improves genomic stability and enhances metabolic efficiency [41]. Beyond this, SIRT1 also modulates proteostasis, mitochondrial function, nutrient-sensing pathways and inflammation [41]. mTOR is implicated in many of the processes that are associated with aging, including cellular senescence, immune responses, cell stem regulation, autophagy, mitochondrial function and proteostasis [42].

As a source of ROS, XOR can cause cellular alterations, such as peroxidation of membrane lipids, DNA damage and protein oxidation, impairing mitochondrial function and leading to apoptosis. In addition, it has been demonstrated that, in HepG2 cells, uric acid induces superoxide generation and mitochondrial dysfunction [43] and it may play a key role in AMPK downregulation [44]. These data suggest that uric acid produced by XOR activity could lower AMPK/SIRT1 levels and augment the mTOR and IGF pathways. However, recent evidences, both in in vitro and in vivo, demonstrate that AMPK activation can be induced by XOR via the nitrate–nitrite–NO pathway [45], suggesting a dual role of XOR in regulating this pathway.

Both in vitro and in vivo experiments show that XO induces mitochondrial ROS via the PI3K–AKT–mTOR pathway. XO-derived ROS, but not uric acid, cause IL1β release and XO inhibition with febuxostat results in impaired IL1β secretion. This pathway represents a mechanism for regulating NLRP3 inflammasome activation and inflamm-aging [38].

Beyond the effects reported above, it can be hypothesized that XOR-induced oxidative stress may activate other pathways regulating cellular senescence, in particular the p53/p21 and p16/pRb pathways. In fact, it is well known that these pathways increase in senescent cells, being activated by telomere dysfunction, DNA damage and chromatin disruptions, cellular and mitogenic stresses [46].

It has been demonstrated that NO produced by XOR nitrite reductase activity inhibits the proliferation of smooth muscle cells in vessels via p21Waf1/Cip1 signaling, increasing CDK1 protein levels. The addition of allopurinol, however, inhibits these effects, demonstrating XOR’s involvement [47].

In alveolar endothelial cells, XOR-induced apoptosis upstreaming p53 results in DNA double-strand breaks and activation of the ataxia telangiectasia mutated (ATM) enzyme, which phosphorylates histone H2AX and upregulates p53 [48].

Several pharmacological agents are currently being studied for their anti-aging effects. Among these compounds, the most promising include metformin, rapamycin, resveratrol and spermidine [49]. Evaluating how these drugs can regulate XOR and its products is important in understanding the role of XOR in aging.

The effects of metformin on XOR activity were evaluated in a clinical study in type 2 diabetic patients, in which plasma XOR activity was significantly decreased after administration of metformin. Preliminary in vitro studies showed that therapeutic as well as higher metformin doses significantly inhibited XO activity, indicating that metformin may directly influence the activity of XO [50]. Since XO is an essential source of ROS, and large amounts of oxidase should be produced following conversion of XDH into XO in hypoxic diabetic tissues, it can be hypothesized that metformin reduces hypoxia and toxic tissue damage probably through the inhibition of XO activity [50].

The effects of the mTOR inhibitor rapamycin (Sirolimus) on serum uric acid levels were studied in renal transplant patients. Immunosuppressive therapy with calcineurin inhibitors elevated uric acid levels, whereas the switch to a Sirolimus-based regimen reversed the rise in serum uric acid levels in these patients [51]. These results, albeit indirectly, suggest that inhibition of the mTOR pathway is important for reducing XOR activity.

Resveratrol inhibits XO in vitro in a dose-dependent manner with the half inhibitory concentration (IC_50_) of 96.7 μM. The inhibition of XO is competitive with a dissociation constant Ki of 9.7 μM [52].

Spermine inhibits the Fe(III)/xanthine oxidase forming an unreactive chelate with iron, thus preventing the generation of hydroxyl radicals by the Haber–Weiss reaction [53]. It is noteworthy that some polyamines, such as spermidine and its derivative spermine, have recently emerged as promising anti-aging agents [49].

**Figure 3:**
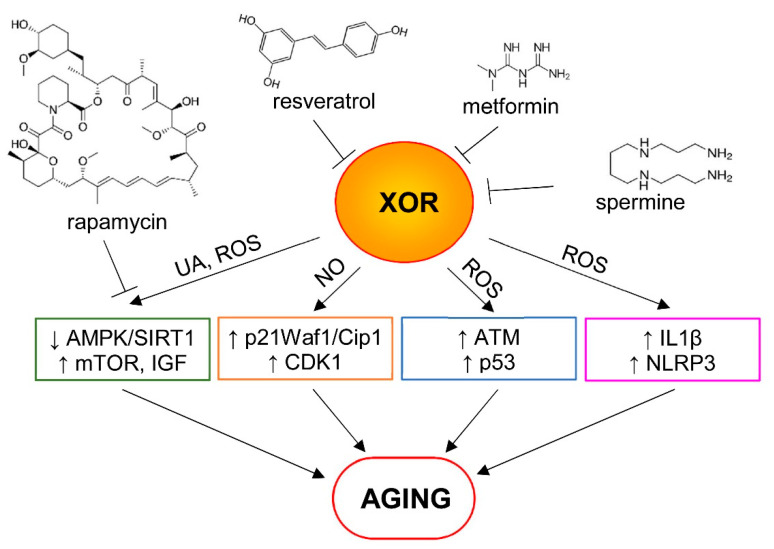
Some proposed mechanisms of aging involving xanthine oxidoreductase (XOR) and its products. Uric acid (UA) and reactive oxygen species (ROS) can play a role in the downregulation of AMP-activated protein kinase/sirtuin-1 (AMPK/SIRT1) levels and in the upregulation of the mTOR and insulin/insulin-like growth factor (IGF) pathways. Nitric oxide (NO) produced by XOR nitrite reductase activity can inhibit cell proliferation, activating p21Waf1/Cip1 signaling and increasing the cyclin-dependent kinase 1 (CDK1) protein level. In addition, XOR-derived ROS can activate the ataxia telangiectasia mutated (ATM) enzyme, which upregulates p53, and causes IL1β release, as well as NLRP3 inflammasome activation and inflamm-aging. Pro-aging effects of XOR can be reduced by some pharmacological agents that inhibit the mTOR pathway (e.g., rapamycin) or interfere with XOR activity (e.g., resveratrol, metformin and spermine), as described in the text.

In Figure 3, some mechanisms of XOR pro-aging effects and the anti-aging effects of the above described pharmacological agents are proposed.

### 4.2. In Vitro Experiments

A series of in vitro experiments shows the effect of ROS produced by the xanthine/xanthine oxidase (X/XO) system in relation to aging.

After exposure to the X/XO system, cultured human dermal fibroblasts show an increased expression of elastin mRNA, which mimics the elastin deposition reported in solar elastosis, suggesting a role for XOR-derived ROS in skin photo-aging [54]. The contribution of ROS, including those produced by XOR activity, to the aging process in skin is higher than in any other organ both in the epidermis and in the dermis, because of the exposition to oxidative stress due to extrinsic factors, mainly UV irradiation [55].

The oxidative stress induced by either H_2_O_2_, XOR activity or minimally oxidized LDL suppresses the osteogenic differentiation of embryonic fibroblast cell lines and mouse primary bone marrow stromal cell cultures by inhibiting the Hedgehog signaling pathway. These results could, in part, explain the role of oxidative stress in the aging of osteoprogenitor cells [18].

The dysregulation of cholesterol homeostasis may result as toxic for cells, in particular for brain cells. The X/XO free radical generating system induces the apoptotic death of human neuroblastoma cell cultures; it also reduces the cellular cholesterol levels, inducing the expression of the genes of the cholesterol biosynthesis pathway, which are associated with the risk for Alzheimer’s disease [56]. On the other hand, Alzheimer’s disease and other degenerative cerebral pathologies derive from the abnormal aggregation of peptides that occurs in amyloidosis. However, the mild oxidative stress generated by X/XO addition causes a reduction in beta-secretase activity and consequently the level of the amyloidogenic protein Aβ [57]. This protective role of the X/XO system is due to a partial inhibition of cathepsin B, which possess a beta-secretase activity [58].

The accumulation of advanced glycation end-products (AGE) is recognized as one of the mechanisms that speed up the aging process by aggravating cell and tissue damage and anticipating the end illness. In vitro studies have ascertained that X/XO-derived ROS are able to accelerate the AGE presence, indicating the relevance of ROS in inducing AGE constitution [59]. These results are in agreement with the correlation reported between the serum level and activity of XOR and the AGE formation in diabetic patients [60].

These in vitro experiments with the X/XO system corroborate the hypothesis that XOR activity can contribute to the progressive changes leading to the aging of skin and osteoprogenitor cells and suggest a role for XOR-derived ROS in degenerative cerebral diseases and diabetes.

### 4.3. Animal Studies

A rat model of neuropathic pain was used to study the influence of oxidative stress and NO on healing after sciatic hurt in young and old animals. The spontaneous recovery is delayed with aging. The level of XOR activity and lipid hydroperoxides in the sciatic nerve rises after injury and is higher in old than in young rats. The antioxidant tirilazad mesylate reduces the level of lipid hydroperoxides and affects hyperalgesia by prolonging or alleviating it, depending on an early or late administration of the drug, respectively. The hyperalgesia is also alleviated by using a neuronal NO synthase inhibitor, implying that NO contributes to the delay of nerve repair in old animals. These results suggest that XOR-derived ROS may have a positive role in a precocious phase after the injury by initiating tissue repair, but also a negative effect in a later phase by delaying the recovery of injured nerves [61].

XOR activity in the rat heart grows approximately 25 times with age compared to new-born to 15-week-old animals [62]. Accordingly, the levels of XOR expression and activity in rat kidney increase with age up to 18 months, then slightly decrease in the following 6 months, whereas the XO/XDH ratio and ROS production progressively increase up to 24 months, possibly contributing to the elevation of aging-related oxidative stress [63]. At some contrast, XOR expression shows no differences in rat coronary arterioles by comparing young and aged animals, although the aging-induced phenotypic changes contribute to the development of oxidative stress that impairs functional vasodilation [64].

A higher blood pressure and impaired renal function are reported in aging rats as compared to young animals. In the aorta of aging rats, these differences are associated with increased free radical generation, XOR expression and activity, but not NAD(P)H activity, suggesting that the vascular aging-associated oxidative stress is XOR-dependent [65]. In addition, XOR expression and activity are significantly higher in the aortic wall and skeletal muscle of old than in those of young rats [66]. The superoxide formation in mesenteric arteries isolated from young and old rats is proportional to blood pressure and high blood pressure induces a higher increase in O_2_^•−^ production in old than in young rat vessels. Confocal microscopy shows that O_2_^•−^ is generated mostly by the endothelium but also by vascular smooth muscle cells. Upregulation of XOR but not of NAD(P)H expression is reported in aged vessels, suggesting that XOR-derived O_2_^•−^ production may contribute to the aging process [67].

The corneal epithelium of aged rabbits has a lower activity level of scavenger enzymes such as superoxide dismutase, glutathione peroxidase and catalase as compared to young adult animals, whereas the activity level of XOR is almost unchanged. The imbalance between antioxidant and pro-oxidant enzymes leads to presume an increased susceptibility of aged corneas to oxidative stress [68].

XOR activity is greater in the skeletal muscle of aged than young mice and increases after electrical stimulation in the muscle of both young and old mice as compared to controls. XOR inhibition by allopurinol reduces oxidative stress and improves muscle function by increasing the contraction force in aged mice [69].

Mouse hematopoietic stem cells from bone marrow of aged mice, in comparison to cells from young animals, show an increase in the levels of pro-inflammatory markers and in intracellular O_2_^•−^, H_2_O_2_, NO and peroxynitrite/hydroxyl, together with a higher level of DNA damage and apoptosis. These cells show also a significantly shorter telomere length, a lower expression of plasticity markers and reduced activity of antioxidant enzymes. Major sources of ROS generation in these hematopoietic stem cells are mitochondria, NAD(P)H, CYP450 and XOR. However, the contribution of XOR activity to ROS production is relevant only in cells from aged animals [70].

XOR expression and activity increase with age in mouse liver, kidney and thymus. This age-related XOR increment is missed in long-lived animals. In peritoneal leukocytes from old mice, XOR contributes to the generation of O_2_^•−^, together with NAD(P)H oxidase, and also produces H_2_O_2_ [71]. In agreement with these results, XOR activity and lipid peroxidation increase with age in the liver, cerebral cortex and plasma of female mice, suggesting a crucial role for XOR-induced oxidative stress in aging [72]. In addition, XOR expression and activity as well as ROS production and lipofuscin accumulation increase with age, together with an impaired immune function in murine peritoneal macrophages except for leukocytes from long-lived mice. These results suggest that the XOR-derived oxidative stress contributes to immunosenescence [73].

Mice fed on an obesogenic diet show an increased XO activity in spleen together with a higher level of oxidative stress and lower level of cellular immune response as compared to the control group. These alterations support the hypothesis that obesity-induced oxidative stress is associated with early immunosenescence [74].

Female mice with haploinsufficiency (hemizygous; HZ) of the tyrosine hydroxylase (TH) enzyme, responsible of catecholamine production, show premature deterioration of sensorimotor abilities and exploratory capacity together with lower immunological responses and higher oxi-inflamm-aging parameters than their wild type littermates. The increased XOR activity in mouse peritoneal leukocyte lysate contributes to oxidative stress and early aging, which reduce their lifespan [75].

Hyperuricemia is associated with aging and contributes to endothelial cell dysfunction and senescence by activating the renin-angiotensin system and impairing NO production. Western diet-fed mice show increased aortic XOR activity and oxidative stress associated with impaired aortic vasodilation that is suppressed by XOR inhibition with allopurinol [76].

The reported animal studies suggest that the progressive increase in the XOR level with age could induce oxidative stress influencing nerve recovery, artery vasodilation, muscle efficiency, hematopoietic stem cells survival and immunosenescence.

### 4.4. Clinical Reports

Numerous animal studies report an increased myocardial XOR activity in heart failure models and show the beneficial effects of XOR inhibition with allopurinol, suggesting a role for XOR-derived ROS in the pathogenesis of chronic heart failure. However, controversial results are obtained by clinical studies that include treatment with XOR inhibitors in the attempt to reduce cardiomyopathy mortality [77].

By comparing old to young healthy subjects, impaired artery dilatation appears with aging, but the level of XOR expression in endothelial cells collected from an antecubital vein is the same in the two groups. In addition, no differences in artery dilation are induced by XOR inhibition with the administration of allopurinol [78]. However, different conclusions derive from the evaluation of age-related vascular dysfunction in the gastric submucosal artery obtained from young and elderly patients after gastrectomy. Only subjects without cardiovascular, renal or diabetic diseases were included in this study. In comparison to young arteries, a thickening of the vessel wall and reduced vasodilation responses are observed in old arteries. In addition, increased XOR expression and malondialdehyde and H_2_O_2_ content are reported with aging together with a decreased expression of superoxide dismutase and glutathione peroxidase, suggesting that increased oxidative stress is associated with decreased antioxidant defense in senescence [79].

Patients operated on for senile cataract show that XOR activity in serum is positively correlated with the patient’s age, while it is negatively correlated in the lens. The activity of XOR both in the serum and in the lens, as well as the concentration of conjugated dienes in the lens, are higher in diabetics than in non-diabetic subjects and are positively correlated with the HbA1C blood concentration. These results suggest that an increase in XOR activity due to hyperglycemia can accelerate the onset of cataracts [80].

Endothelial XO is one of the sources of ROS and RNS that are responsible for the presence of damaged proteins into the circulation. The excessive production together with inadequate repair or removal of these oxidized molecules favors their accumulation, thus contributing to the aging process [81].

A 10-year retrospective observational study including 3593 patients of an elderly rehabilitation unit showed a significantly higher improvement in the allopurinol-treated (102 patients) than in the control group as measured by the Barthel score to evaluate skeletal muscle function. These results confirm the implication of XOR-induced oxidative stress in the development of sarcopenia [82].

Type 2 diabetic patients are more subjected to develop sarcopenia in the elderly than in non-diabetic old people. In a cross-sectional study, designed to investigate both oxidative stress and antioxidant status, plasma XO is found significantly higher in sarcopenic old diabetic patients, suggesting a role for XOR activity in the pathogenesis of sarcopenia in diabetes [83].

A long-lasting debate still not completely resolved is whether hyperuricemia represents a cause or simply a marker of aging-associated diseases. In a longitudinal study with 937 healthy Taiwanese aged people with uricemia in the normal range, a significant correlation was observed between the level of serum uric acid level and the 10-year cardiovascular Framingham risk score [84]. Similarly, the recent InCHIANTI study enrolling 947 Italian subjects suggested that the lowest nine-year cardiovascular risk for an apparent healthy elder population is related to serum uric acid around 4 mg/dL, while the mortality risk significantly increases for values above 4.3 mg/dL, which is far below the upper limit of the normal range [85]. These results are not sufficient to recommend the use of drugs to lower uricemia in asymptomatic aged people. However, they suggest to carefully check the serum level of uric acid in the contest of other parameters of cardiovascular risk.

Recent reviews and meta-analysis studies focused on the role of uric acid in dementia/cognitive impairment related to senescence, suggesting that uric acid has a protective action in Alzheimer’s disease and Parkinson’s dementia (hydrophilic antioxidant properties), while hyperuricemia may have a negative influence during vascular dementia (stroke, small vessel cerebrovascular disease) [86,87].

**Figure 4:**
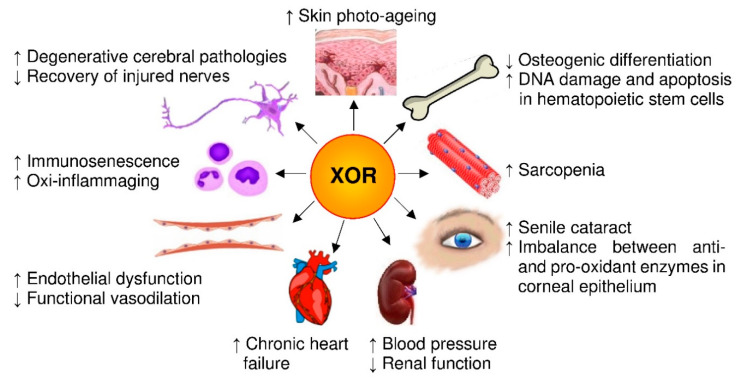
Pro-aging effects of xanthine oxidoreductase (XOR) products. Senescence is associated with an XOR-dependent oxidative stress that affects the cells of many organs and systems, as exemplified in the image. In addition, this pro-oxidant state contributes to the formation of advanced glycation end-products, thus aggravating the conditions of diabetic patients.

Pro-aging effects of XOR products are schematized in Figure 4. Controversial results are obtained with clinical studies. However, XOR activity and products may contribute to vascular aging and, in the presence of hyperglycemia, may accelerate the onset of cataracts. In addition, XOR-derived oxidative stress appears to promote senile sarcopenia, especially in the diabetic population.

## 5. Conclusions

A considerable amount of evidence highlights the crucial role of oxidative stress in the aging process following both the progressive increase in ROS and RNS production and the reduction in the efficiency of antioxidant scavenger systems. If the resulting balance between these metabolic processes leads to an excessive formation of free radicals, the consequence is the pathological modification of glucides, lipids, proteins and DNA. The accumulation of these altered molecules strongly contributes to the progressive loss of physiological functions and to the increase in chronic inflammatory diseases, which both characterize senescence. This justifies the oxi-inflamm-aging name given to the prevailing transformation modality that occurs in the body of the elderly in contrast to centenarians, who show an antioxidant profile and represent a group of successfully aged people. In addition, the oxidative stress contributes to the senescence of multipotent stem cells, thus impairing their ability to repair injured tissues.

XOR is one of the sources of reactive species and it is present in all cell types, although in most cases with a low level of activity and acting mainly as dehydrogenase. In various types of pathological conditions, it has been reported that XOR activity produces O_2_^•−^ and H_2_O_2_ from which highly cytotoxic hydroxyl radicals can generate. Under hypoxic and low-pH conditions, XOR can produce NO from which highly cytotoxic ONOO^−^ can be derived. Furthermore, the uric acid produced by the activity of XOR can give rise to dangerous free radicals. These observations, together with the increase in both XOR activity and oxidative stress associated with aging, suggest that XOR products contribute to the senescence process. For these reasons, XOR inhibition through specific drugs is recommended by many authors, for example, in cardiovascular pathology [88].

Hyperuricemia is associated with most aging-related diseases and a growing number of research points to urate-lowering therapy to verify the role of uric acid, especially in kidney and cardiovascular diseases. A retrospective epidemiological cohort study was conducted for eight years including 2690 patients with chronic kidney disease and baseline serum uric acid level greater than 7 mg/dL. These patients received urate-lowering therapy during follow-up, mainly consisting of treatment with allopurinol. Among these subjects, 42% achieved a serum uric acid level below 6 mg/dL and experienced a 30% improvement in the estimated glomerular filtration rate [89]. A recent review analyzes the role of hyperuricemia and gout in hypertension and cardiovascular disease and the effect of urate-lowering therapy. Allopurinol shows cardioprotective benefits due mainly to the decrease in the serum level of uric acid and the consequent decrease in inflammation and oxidative stress [90].

These reports suggest the use of XOR inhibitors in the case of asymptomatic hyperuricemia associated with chronic kidney disease to delay the onset of renal failure or to reduce the risk of adverse cardiovascular events in patients with gout. However, urate-lowering therapy with XOR inhibitors, recombinant uricase or uricosuric drugs can also have harmful outcomes that require caution. When the serum uric acid level is in the lower quartile, higher all-cause mortality was observed, suggesting a U-curve type of relationship between uric acid and mortality. Low serum urate is associated with worse outcomes in neurological conditions, such as Parkinson’s and Huntington’s diseases and amyotrophic lateral sclerosis, suggesting a neuroprotective role of uric acid as an antioxidant [91].

It should also be considered that NO, O_2_^•−^ and H_2_O_2_ derived from XOR have an essential role in the regulation of endothelial functions and vascular tone. In fact, reactive oxygen and nitrogen species have an indispensable physiological role as a second messenger to modulate different cellular outcomes and vascular responses. This ambivalent behavior of some ROS and RNS, as well as uric acid, depends mainly on the concentration they reach, leading to oxidative stress only if their level is much higher than normal. All together, these motivations justify the inconsistency of the results obtained with XOR inhibitors and the caution in assessing the appropriateness of their clinical use in asymptomatic hyperuricemia.

The development of new drug strategies to fine-modulate XOR activities, together with a careful evaluation of the patient’s clinical picture, could, in the future, allow a better management of aging-related chronic diseases.
